# Basal Body Protein TbSAF1 Is Required for Microtubule Quartet Anchorage to the Basal Bodies in *Trypanosoma brucei*

**DOI:** 10.1128/mBio.00668-20

**Published:** 2020-06-09

**Authors:** Xiaoduo Dong, Teck Kwang Lim, Qingsong Lin, Cynthia Y. He

**Affiliations:** a Mechanobiology Institute, National University of Singapore, Singapore; b Department of Biological Sciences, National University of Singapore, Singapore; c Centre for BioImaging Sciences, National University of Singapore, Singapore; University of Georgia

**Keywords:** Spef1/CLAMP, microtubule quartet, MtQ, basal bodies, flagellar pocket, *Trypanosoma brucei*

## Abstract

Sperm flagellar protein 1 (Spef1, also known as CLAMP) is a microtubule-associated protein involved in various microtubule-related functions from ciliary motility to polarized cell movement and planar cell polarity. In Trypanosoma brucei, the causative agent of trypanosomiasis, a single Spef1 ortholog (TbSpef1) is associated with a microtubule quartet (MtQ), which is in close association with several single-copied organelles and is required for their coordinated biogenesis during the cell cycle. Here, we investigated the interaction network of TbSpef1 using BioID, a proximity-dependent protein-protein interaction screening method. Characterization of selected candidates provided a molecular description of TbSpef1-MtQ interactions with nearby cytoskeletal structures. Of particular interest, we identified a new basal body protein TbSAF1, which is required for TbSpef1-MtQ anchorage to the basal bodies. The results demonstrate that MtQ-basal body anchorage is critical for the spatial organization of cytoskeletal organelles, as well as the morphology of the membrane-bound flagellar pocket where endocytosis takes place in this parasite.

## Abstract (precis)

Trypanosoma brucei contains a large array of single-copied organelles and structures. Through extensive interorganelle connections, these structures replicate and divide following a strict temporal and spatial order. A microtubule quartet (MtQ) originates from the basal bodies and extends toward the anterior end of the cell, stringing several cytoskeletal structures together along its path. In this study, we examined the interaction network of TbSpef1, the only protein specifically located to the MtQ. We identified an interaction between TbSpef1 and a basal body protein TbSAF1, which is required for MtQ anchorage to the basal bodies. This study thus provides the first molecular description of MtQ association with the basal bodies, since the discovery of this association ∼30 years ago. The results also reveal a general mechanism of the evolutionarily conserved Spef1/CLAMP, which achieves specific cellular functions via their conserved microtubule functions and their diverse molecular interaction networks.

## INTRODUCTION

Sperm flagellar protein 1 (Spef1) is a microtubule (MT)-associated protein first described in mouse sperm flagellum (1). Dougherty et al. independently identified the expression of the same protein in mouse pillar cells (2) and demonstrated its ability to bind and bundle microtubules via a calponin-homology domain. Spef1 was therefore also named CaLponin-homology And Microtubule-associated Protein (CLAMP). Further functional genomics analyses (3) identified Spef1 as a conserved component of organisms containing motile flagella/cilia, including the parasitic protozoan Trypanosoma brucei. RNA interference (RNAi) depletion of TbSpef1, the single Spef1/CLAMP orthologue in T. brucei, led to severe motility defects, supporting its role in flagellar motility. The flagellar motility function of Spef1 is further strengthened by recent work, where mouse Spef1 is localized to the central pair of ependymal cilia and is found essential for central pair biogenesis and ciliary motility (4). In addition to ciliary motility, studies in *Xenopus* revealed a broad role of Spef1/CLAMP in cell polarity, likely through its interaction with polarity determining factors (e.g., the Par complex) and its effect on microtubule dynamics (5, 6). It thus appears that Spef1/CLAMP, despite their conserved molecular functions on microtubule association and bundling, have distinct cellular functions in different organisms and/or cell types, possibly through Spef1/CLAMP interaction with different cellular components.

T. brucei is a member of the *Trypanosomatidae* family that contains many parasitic flagellates responsible for various human, animal, and plant diseases (7). Like all trypanosomatids, T. brucei contains a long, motile flagellum essential for cell motility and other cellular functions (see Fig. S1 in the supplemental material) (8). The flagellum is nucleated from the basal bodies, which are physically attached to the kinetoplast that contains mitochondrial DNA through a tripartite attachment complex (TAC) (9, 10). The flagellar pocket, which is a plasma membrane invagination at the base of the flagellum, is responsible for most of the endocytosis/exocytosis activities in the cell. The flask-shaped flagellar pocket is delimited by the basal bodies on one end and a horseshoe-shaped flagellar pocket collar (FPC) on the other (11). Between the basal bodies and the FPC, a set of four specialized microtubules known as the microtubule quartet (MtQ) originates from the basal bodies and wraps around the flagellar pocket membrane, likely supporting its distinct flask shape (12). Each of the above-mentioned structures is present as a single copy in each cell. During the cell cycle, these structures duplicate and divide in a highly coordinated fashion, following a strict temporal and spatial order (11, 13). This coordination is likely mediated by the physical tethering of these structures to one another (14, 15). Some of the tethering mechanisms have been well studied in T. brucei, for instance, the TAC that links the kinetoplast to the basal bodies. More recent work revealed interaction between the FPC and a hook complex (HC) in the bilobe region located at the base of the flagellum via a microtubule-binding protein FPC4 (16). This interaction explains the close association between the FPC and the HC during their replication and division. However, little is known about the molecular nature of other interorganelle connections.

10.1128/mBio.00668-20.1 FIG S1 Schematic representation of T. brucei cellular architecture. A series of single-copied organelles are present in a T. brucei procyclic cell, from posterior to anterior, including kinetoplast, basal body, flagellar pocket, MtQ, FPC, FAZ filament, bilobe, Golgi apparatus, and flagellum. These organelles are likely tethered to form a continuous network to facilitate their coordinated biogenesis. The best characterized organelle tether is the tripartite attachment complex (TAC) that links the kinetoplast to the basal bodies. The whole cell membrane, with the exception of the flagellar pocket membrane, is subtended by arrays of stable subpellicular microtubules. Download FIG S1, JPG file, 0.7 MB. Copyright © 2020 Dong et al. 2020 Dong et al. This content is distributed under the terms of the Creative Commons Attribution 4.0 International license.

In our previous study (14), TbSpef1 was found to be associated with the proximal end of the MtQ between the basal bodies and the FPC. In cells depleted of TbSpef1 by RNAi, perturbation of biogenesis was observed with the MtQ and MtQ-associated structures such as the FPC, the bilobe complex that contains a TbCentrin4-positive arm and the TbMORN1-positive HC (17–19), and the flagellum attachment zone (FAZ) that is required for flagellum-driven cell motility and cell morphogenesis (20–28). These results also suggested a key role of MtQ in the organization and biogenesis of cytoskeletal organelles in this parasite. Since TbSpef1 is the only protein known to date that specifically localizes to the MtQ (29), further studies of TbSpef1 and its interacting partners will help to understand the function of MtQ in T. brucei.

In this study, we used BioID, a proximity-dependent biotinylation method (30), to identify neighboring proteins of TbSpef1. A previously uncharacterized basal body protein TbSAF1 (TbSpef1-Associated Factor 1, encoded by Tb927.7.4370) was found between the probasal and mature basal bodies. In cells depleted of TbSAF1, the MtQ was found disassociated from the basal bodies at both light and electron microscopic levels. The dissociation was also evident by the changed position of MtQ-associated organelles such as the FPC, HC/bilobe, and FAZ relative to the basal bodies, as well as morphological changes of the flagellar pocket. The results confirmed the conserved molecular activity of Spef1/CLAMP in microtubule binding and bundling and posited a general cellular mechanism of Spef1/CLAMP by associating microtubules with different cellular components to achieve different cellular functions.

## RESULTS

### TbSpef1 binds and bundles microtubules *in vitro*.

Both mouse and *Xenopus* Spef1/CLAMP bind microtubules (2, 5). Overexpression of mouse Spef1 in COS-7 cells has also been shown to bundle and stabilize microtubules *in vivo* (2). TbSpef1, encoded by Tb927.4.3130, shows an overall similarity (positive substitutions) of 68% to mouse Spef1 and 49% to *Xenopus* Spef1/CLAMP. To examine whether TbSpef1 can bind microtubules, purified His-TbSpef1 was incubated with *in vitro*-polymerized and paclitaxel (originally named taxol)-stabilized microtubules (see Fig. S2A in the supplemental material). In the absence of microtubules, TbSpef1 was a soluble protein, remaining in the supernatant after high-speed centrifugation. In the presence of microtubules, TbSpef1 cosedimented with the microtubules, confirming their interaction. The binding saturated when the molar ratio of TbSpef1 to tubulin dimer was >1, suggesting that each α/β-tubulin dimer can bind at least one TbSpef1 molecule.

10.1128/mBio.00668-20.2 FIG S2 TbSpef1 binds and bundles microtubules *in vitro.* (A) *In vitro*-polymerized and paclitaxel-stabilized microtubules were incubated with purified TbSpef1 at indicated concentrations for 30 min, and microtubules were precipitated by centrifugation. TbSpef1 binding to the microtubules was evaluated by cosedimentation with polymerized microtubules by immunoblotting. (B) Rhodamine-labeled microtubules were incubated with increasing concentrations of purified TbSpef1 and imaged with fluorescence microscopy. Inset shows enlarged view of the boxed region containing unbundled, single microtubules. (C) Polymerized microtubules without or with purified TbSpef1 mixed at 1:1 ratio (10 μM each) were negatively stained and imaged by transmission electron microscopy. (D) Tubulin (15 μM) was polymerized at 35°C for 20 min with or without purified His-TbSpef1 (2.5 μM). Polymerized MTs were then subjected to cold treatment at 4°C for 30 min. (E) Quantification of MTs fluorescence in different conditions shown in panel D. The results are shown as a box-and-whisker plot, with the box showing the 25th to 75th percentiles, the whiskers showing the 5th to 95th percentiles, and the bar inside the box representing the mean. ****, *P* < 0.0001. Download FIG S2, JPG file, 2.3 MB. Copyright © 2020 Dong et al. 2020 Dong et al. This content is distributed under the terms of the Creative Commons Attribution 4.0 International license.

Microtubule bundles could be detected when TbSpef1 was added into the *in vitro*-polymerized rhodamine-labeled microtubules (Fig. S2B), even at a low molar ratio of TbSpef1 to tubulin dimer (1:20). More and larger MT bundles were formed with increasing TbSpef1 concentration. Under transmission electron microscopy (TEM), the MT bundles were found to contain two to five MTs that are laterally joined together (Fig. S2C). Importantly, formation of TbSpef1-induced MT bundles was reversible by shifting the reaction to 4°C (Fig. S2D and E), suggesting that these structures were unlikely formed due to protein aggregation. Together, these results showed that TbSpef1 can bind and bundle microtubules, similar to that reported with mouse and Xenopus Spef1/CLAMP (2, 5). MT binding and bundling is thus an evolutionarily conserved molecular function of Spef1/CLAMP from protozoa to vertebrates.

### TbSpef1 BioID.

The different cellular locations and functions of Spef1/CLAMP reported so far in different cell types raised the possibility that Spef1/CLAMP achieves various functions by associating microtubules to different cellular components. To further understand the cellular mechanisms of TbSpef1, we used BioID, a proximity-dependent screen for protein-protein interactions (30–32), to identify TbSpef1 neighboring proteins. The promiscuous biotin ligase BirA* was inserted upstream or downstream of an endogenous TbSpef1 allele, allowing stable endogenous expression of recombinant BirA*-TbSpef1 protein (33, 34). The expression of BirA*-TbSpef1 and specific biotinylation of proteins near the TbSpef1-containing MtQ were confirmed by immunofluorescence assays and immunoblotting (Fig. S3A and B).

10.1128/mBio.00668-20.3 FIG S3 Validation of proximity-dependent biotinylation by BirA*-TbSpef1. Control (WT) and BirA*-TbSpef1 cells were incubated with 5mM biotin for 24 h. (A) Cells were labeled with anti-TbSpef1 (green) and Alexa Fluor 594-conjugated streptavidin (magenta) for immunofluorescence microscopy. (B) Biotinylated proteins were affinity purified with streptavidin beads and assayed by immunoblots probed with anti-TbSpef1 and streptavidin-HRP prior to LC/MS analyses. T, total cell lysate; S, supernatant after extraction with 1% NP-40 and 0.4% SDS (see Materials and Methods); U, unbound fraction; AP, proteins eluted from streptavidin beads. Download FIG S3, JPG file, 0.9 MB. Copyright © 2020 Dong et al. 2020 Dong et al. This content is distributed under the terms of the Creative Commons Attribution 4.0 International license.

Biotinylated proteins were captured by streptavidin-beads and analyzed by liquid chromatography-tandem mass spectrometry (LC-MS/MS). Three independent experiments were performed, with two performed on N-terminally tagged BirA*-TbSpef1 fusion and one on a C-terminal fusion. A total of 33 protein candidates were specifically identified in cells expressing BirA*-TbSpef1 fusions but absent in control samples (Table 1). Among these, 11 high-confidence candidates, including TbSpef1 itself, were identified in at least two of three BioID experiments (Table 1). A putative MtQ/bilobe protein (Tb927.4.4180) and a known FAZ protein FAZ10 (Tb927.7.3330) (35) were also found among the high-confidence candidates. Eight BioID candidates were analyzed further; all of them were previously uncharacterized proteins from the high-confidence list (Table 1).

**TABLE 1:** High-confidence TbSpef1-neighboring proteins identified through BioID

Gene ID^*a*^	Protein description	Protein localization		Protein content (%)^*b*^
		Tryptag (36)	This study	
**Tb927.7.4370**	TbSAF1		Basal bodies	2.77/NA/3.02
**Tb927.5.570**	Hypothetical protein, conserved	HC/bilobe region	HC/bilobe region	2.56/1.03/4.02
Tb927.4.4180	SUMO-interacting motif-containing protein	MtQ		1.83/0.93/3.20
**Tb927.10.12720**	Hypothetical protein, conserved	HC/bilobe region	HC/bilobe region	1.50/3.70/0.45
**Tb927.8.8160**	SUMO-interacting motif-containing protein		MtQ	1.20/0.52/2.00
**Tb927.3.2760**	Hypothetical protein, conserved		HC/bilobe region	1.15/NA/2.01
Tb927.4.3130	TbSpef1 (bait protein)	MtQ	MtQ	0.93/1.04/2.41
**Tb927.11.1220**	Hypothetical protein, conserved	MtQ	MtQ	0.90/1.04/0.48
**Tb927.6.1220**	Hypothetical protein, conserved	Basal bodies	Basal bodies	0.88/0.48/0.49
**Tb927.10.250**	Hypothetical protein, conserved	Flagellar proximal	MtQ	0.55/0.96/NA
Tb927.7.3330	FAZ 10	FAZ		0.42/0.74/NA

a Gene IDs indicated in boldface were further characterized in this study.

b Protein contents are from three independent BioID experiments, separated by shills (/). BirA* was tagged at the N terminus of TbSpef1 in the first and second experiments and at the C terminus in the third experiment.

**FIG 1:**
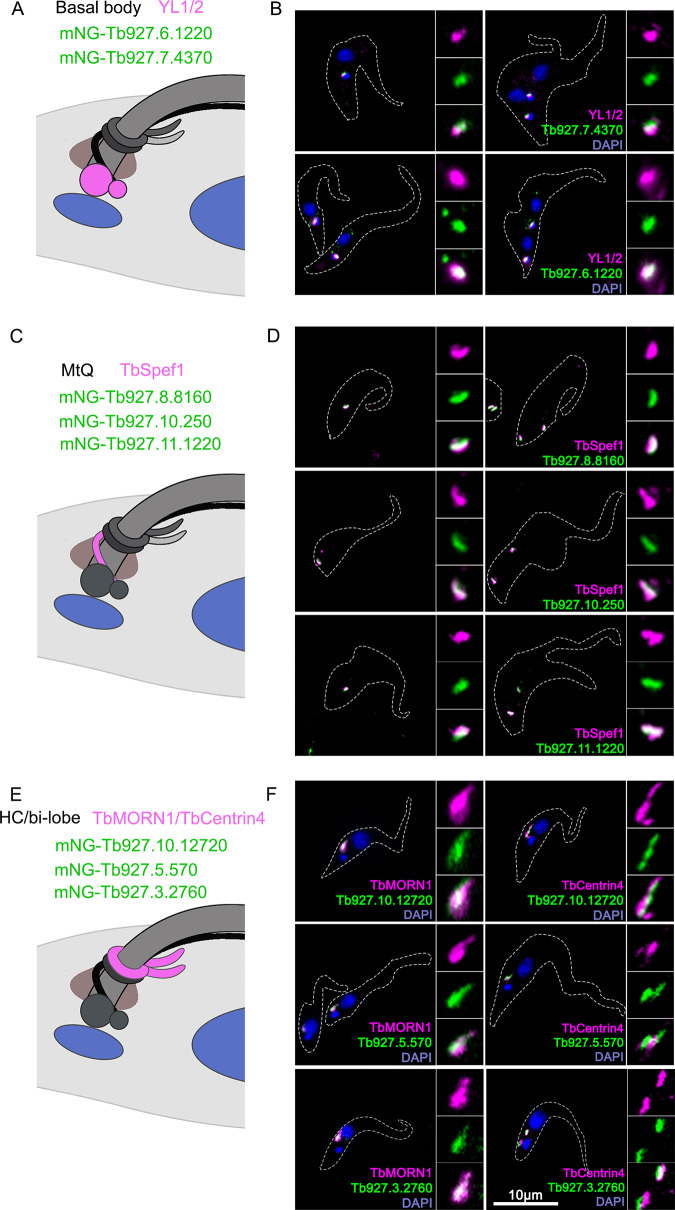
Subcellular localization of selected TbSpef1-BioID candidates. (A, C, and E) Schematic representations of the subcellular organization of the basal bodies, the MtQ, and the HC/bilobe structures at the base of the flagellum, relative to the kinetoplast and the nucleus. (B, D, and F) Selected BioID candidates were endogenously tagged with mNeonGreen (mNG) at the N terminus. Stable transfectants were fixed with cold methanol (B and F) or PFA after extraction with 1% NP-40 and 1 M KCl (D) and stained with DAPI (blue) and basal body, MtQ, or HC/bilobe markers (magenta). For some cell lines (B and D), two cells representative of different cell cycle stages are shown, with each cell demarcated by white dotted lines. The insets show enlarged views of structures in different channels.

All candidates were expressed endogenously with a mNeonGreen (mNG) reporter fused to the N terminus (Fig. 1). Two candidates (Tb927.6.1220 and Tb927.7.4370) were found at the basal body region, partially overlapping with the YL1/2 antibody that stains tyrosinated α-tubulin, as well as RP2 (37). Three candidates (Tb927.8.8160, Tb927.10.250, and Tb927.11.1220) were found at the proximal MtQ region, colocalizing with TbSpef1. Three (Tb927.10.12720, Tb927.5.570, and Tb927.3.2760) were present at the HC/bilobe region, partially overlapping with both TbMORN1 and TbCentrin4 (18, 38). These results support the spatial proximity of the TbSpef1-containing MtQ to both the bilobe region and the basal bodies.

### A TbSpef1 associated protein locates in the region between the probasal and mature basal bodies.

The MtQ originates from a region between the probasal and mature basal bodies (12, 39, 40), but the molecular nature of this association is not yet known. We therefore decided to focus on the two basal body proteins identified in the TbSpef1-BioID screen, encoded by Tb927.6.1220 and Tb927.7.4370, respectively. To evaluate whether their basal body localization depends on the presence of TbSpef1, tetracycline-inducible TbSpef1-RNAi was introduced to cells tagged with mNG endogenously. Upon depletion of TbSpef1, both proteins remained colocalized with YL1/2 (Fig. S4A and B), although basal body segregation was perturbed in some cells, as previously reported (14). The basal body localization of both proteins was therefore independent of TbSpef1.

10.1128/mBio.00668-20.4 FIG S4 Initial characterizations of Tb927.6.1220 and Tb927.7.4370. (A and B) The basal body localization of Tb927.6.1220 and Tb927.7.4370 is not affected by TbSpef1-RNAi. Cells with stable, endogenous expression of mNG-Tb927.6.1220 or mNG-Tb927.7.4370 were induced for TbSpef1-RNAi for 36 h, fixed, and immunolabeled with YL1/2 for the basal bodies. (C and D) RNAi of Tb927.7.4370, but not Tb927.6.1220, led to slower cell doubling. The results are shown as mean doubling numbers ± the SD (*n* = 3). (E) When Tb927.7.4370-RNAi was induced, samples were taken every 24 h, fixed, and stained with DAPI (4′,6′-diamidino-2-phenylindole) to monitor DNA contents by fluorescence microscopy. Multinucleated cells indicate cells with more than two nuclei. Others indicate kinetoplasts, zoids, 1K2N cells, and nK0N cells (*n* > 2). Download FIG S4, JPG file, 1.4 MB. Copyright © 2020 Dong et al. 2020 Dong et al. This content is distributed under the terms of the Creative Commons Attribution 4.0 International license.

Both basal body proteins were further analyzed using tetracycline-inducible RNAi (Fig. S4C and D). Inhibition of Tb927.6.1220 expression produced little growth defect. Inhibition of Tb927.7.4370, however, led to a rapid growth arrest, although the cells continued to proliferate slowly over time. Examination of the DNA contents in Tb927.7.4370-RNAi cells revealed a decrease in cells with one kinetoplast and one nucleus and a slight increase in cells containing duplicated kinetoplasts and nuclei (2K2N), suggesting mildly inhibited cell division (Fig. S4E).

**FIG 2:**
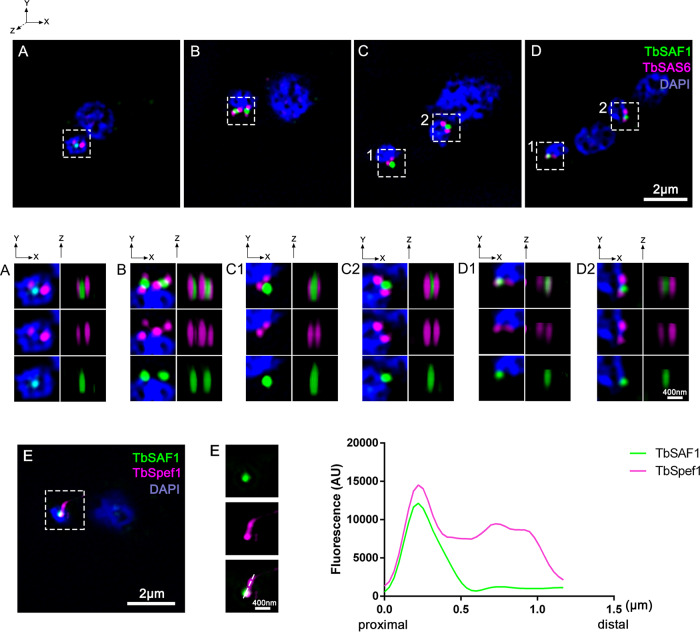
TbSAF1 is localized between pro and mature basal bodies, at the proximal end of TbSpef1-MtQ. (A to D) Cells with endogenous expression of mNG-TbSAF1 were fixed with cold methanol and stained with anti-TbSAS6 antibody. Three-dimensional superresolution images were acquired by 3D-SIM. Cells representative of different cell cycle stages are shown. Enlarged views of the boxed regions in different channels and at different angles are shown below. (E) Cells stably coexpressing mNG-TbSAF1 and mScarlet-TbSpef1 were imaged by 3D-SIM. The association of TbSAF1 with the proximal end of TbSpef1-MtQ is demonstrated by a line plot.

Superresolution three-dimensional structured illumination microscopy (3D-SIM) was performed to examine the exact localization of Tb927.7.4370. A single punctate mNG-Tb927.7.4370 intensity was consistently found in the region between the probasal and mature basal bodies (Fig. 2A to D), marked by anti-TbSAS6 antibodies (41). In addition, the Tb927.7.4370 intensity is in close association with the proximal end of TbSpef1-labeled MtQ (Fig. 2E). The interaction between Tb927.7.4370 and TbSpef1 was also confirmed by reverse BioID using BirA*-Tb927.7.4370 as a bait, where TbSpef1 was identified as a Tb927.7.4370-neighboring protein. We therefore renamed Tb927.7.4370 as TbSpef1-associated factor 1 (TbSAF1) and focused on this protein here.

### TbSAF1 is required for MtQ anchorage to the basal bodies.

**FIG 3:**
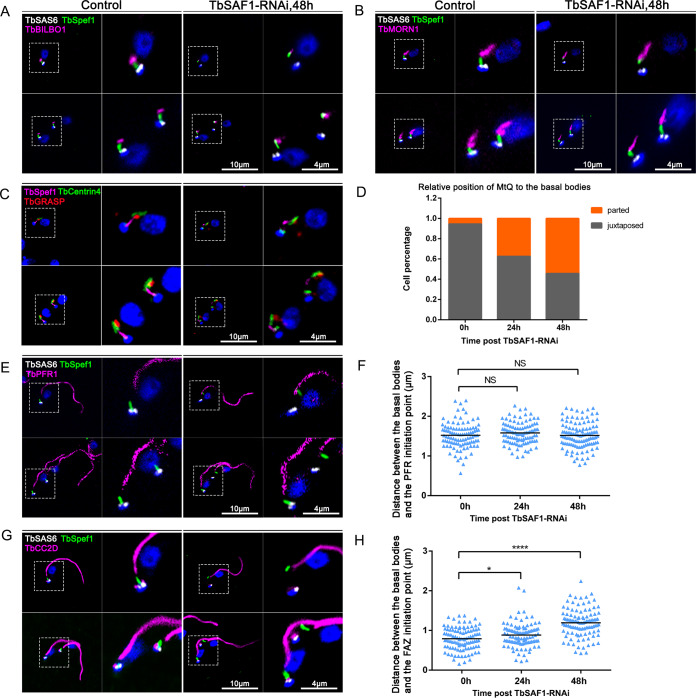
TbSAF1-RNAi alters the position of the MtQ and MtQ-associated FPC, HC/bilobe, and FAZ filament relative to the basal bodies. (A, B, E, and G) TbSAF1-RNAi was induced for 48 h in cells stably expressing YFP-TbSpef1 and mScarlet-TbSAS6. FPC, bilobe, PFR, and FAZ filament were immunostained with anti-TbBILBO1, anti-TbMORN1, anti-PFR1, and anti-TbCC2D antibodies, respectively. (C) TbSAF1-RNAi was induced for 48 h in cells endogenously tagged with mNG-TbSpef1. Basal bodies and bilobe were immunostained with anti-Centrin4 antibodies. The Golgi apparatus was stained with anti-TbGRASP antibodies. (D) The relative position of the TbSpef1-MtQ to the basal bodies was analyzed in control and TbSAF1-RNAi cells. *n* = 100 cells for each condition. (F and H) Quantitation of the distance between the basal bodies and the proximal end of PFR or FAZ. At least 200 cells were measured for each condition. NS, not (statistically) significant; *, *P* < 0.05; ****, *P* < 0.0001.

The respective localization TbSAF1 and TbSpef1 on the basal bodies and the MtQ, as well as their close proximity revealed by BioID and superresolution microscopy, raised an intriguing possibility that they are involved in MtQ association with the basal bodies. To test this possibility, the association of TbSpef1-MtQ to TbSAS6-labeled basal bodies was examined in TbSAF1-RNAi cells (Fig. 3). Normally, the TbSpef1-MtQ appeared as a short rod-like structure, with its proximal end juxtaposed with the basal bodies (Fig. 2E and Fig. 3; see also Fig. S1 in the supplemental material). In TbSAF1-RNAi cells, however, the association between the basal bodies and TbSpef1-MtQ was disrupted, resulting in greater distance between the basal bodies and the TbSpef1-MtQ (Fig. 3D). In addition to TbSpef1, three additional MtQ markers confirmed in this study (Tb927.8.8160, Tb927.10.250, and Tb927.11.1220) all exhibited similar dissociation from the basal bodies upon TbSAF1 depletion (Fig. S5).

10.1128/mBio.00668-20.5 FIG S5 All three new MtQ markers (Tb927.8.8160, Tb927.10.250, and Tb927.11.1220) dissociate from the basal bodies with TbSpef1 upon TbSAF1-RNAi. Tb927.8.8160 (A), Tb927.10.250 (B), and Tb927.11.1220 (C) were endogenously tagged with mNeonGreen; TbSpef1 was endogenously tagged with mScarlet. All cells were extracted with 0.25% NP-40 before fixation with 4% PFA. (D) Schematic representation of TbSpef1 and other MtQ markers detached from the basal bodies upon TbSAF1-RNAi. Download FIG S5, JPG file, 2.3 MB. Copyright © 2020 Dong et al. 2020 Dong et al. This content is distributed under the terms of the Creative Commons Attribution 4.0 International license.

MtQ-associated organelles, including the TbBILBO1-containing FPC (Fig. 3A), the TbMORN1-containing HC (Fig. 3B), and the TbCentrin4-containing bilobe (Fig. 3C), remained associated with the TbSpef1-MtQ in the absence of TbSAF1. Also, like the MtQ, these MtQ-associated structures were positioned further away from the basal bodies. Notably, the Golgi apparatus was also shifted together with the bilobe structure (Fig. 3C), an observation consistent with the previously hypothesized association between these two structures (42).

In control cells, the FPC and HC/bilobe are present at the neck of the flagellar pocket, where the flagellum exits the cell. Along the extracellular portion of the flagellum, a paraflagellar rod (PFR) is assembled parallel to the axoneme, and the FAZ is assembled in the cell subtending the attached flagellum (Fig. S1). The distances between the PFR/FAZ initiation site and the basal bodies were examined and measured in control and TbSAF1-RNAi cells (Fig. 3E to H). Interestingly, while the PFR initiation site did not change, the FAZ initiation site was further away from the basal bodies in TbSAF1-RNAi cells, together with the MtQ, FPC, HC, and bilobe. These results suggest a role of MtQ and associated organelles in determining the assembly site of the FAZ in the cell body, but not the PFR in the flagellum.

**FIG 4:**
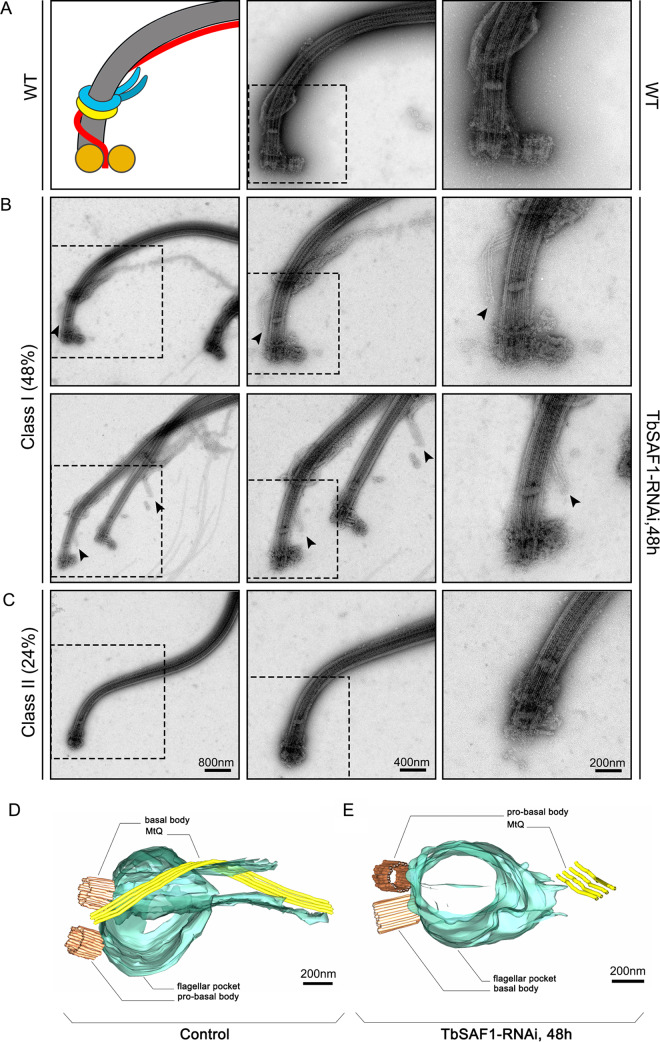
Depletion of TbSAF1 dissociates MtQ, FPC, and HC/bilobe from basal bodies. (A to C) Control (A) and TbSAF1-RNAi cells (B and C) were extracted with detergent (1% NP-40) and high-salt (1 M KCl) buffers, negatively stained, and imaged by TEM. Based on the MtQ and FPC morphology, the TbSAF1-RNAi cells were broadly divided to two phenotypic classes. In each row, images of the same flagellum with increasing magnifications are shown. The arrowheads in panel B indicate the detached MtQ. (D and E) Serial electron tomography of the flagellar pocket region was performed for both control (D) and TbSAF1-RNAi (E) cells. Representative views of the three-dimensional model are shown.

To further validate the TbSAF1-dependent MtQ-basal body association observed by light microscopy, control and TbSAF1-RNAi cells were extracted using detergent and high salt, negatively stained, and examined by transmission electron microscopy (Fig. 4A to C). This method allowed clear visualization of cytoskeletal elements, including the basal bodies (both probasal and mature basal bodies), the flagellum, the MtQ, and the FPC. In control cells, the MtQ originates from between the probasal and mature basal bodies, rotates around the base of the flagellum, and then complexes with the FPC and the HC/bilobe (Fig. 4A). In TbSAF1-RNAi cells, two major classes of mutant phenotypes could be observed (Fig. 4B and C). In class I mutants (∼48%, *n* = 46), all cytoskeletal structures appeared intact. However, the MtQ clearly dissociated and was positioned at a distance from the basal bodies in these cells. The FPC was also positioned farther away from the basal bodies and the basal plate. In class II mutants (∼24%), only the flagellum and the mature basal body could be clearly observed in the extracted cells. MtQ and FPC could not be detected and the probasal bodies were either absent or poorly developed. Since MtQ and FPC were consistently observed in TbSAF1-RNAi cells by immunofluorescence assays (Fig. 3), the absence of these structures in flagellum cytoskeleton may be due to their weakened association with the basal bodies and their subsequent loss during the detergent/high-salt extraction process. Note that such a disassociation between the basal bodies and the MtQ was never observed in control cells extracted, fixed, and imaged under the same conditions.

Dissociation of the MtQ from the basal bodies in TbSAF1-RNAi cells was also confirmed using serial electron tomography. To improve resolution, five continuous sections encompassing the flagellar pocket regions in control and TbSAF1-RNAi cells were imaged by tilting each section around two orthogonal axes. All tilt series were computed, aligned and then combined to produce one single tomogram. The basal bodies, the flagellar pocket and the MtQ were manually annotated and representative views of the 3-dimensional models were shown in Fig. 4D and E. Unlike the control cells, where the MtQ originates from between the basal bodies (Fig. 4D), the MtQ in TbSAF1-RNAi cells was not found near the basal bodies (Fig. 4E). Note that using the current electron tomography method with limited serial sections observed, we could not see exactly where the “loose” MtQ ended in TbSAF1-RNAi cells.

### Depletion of TbSAF1 leads to morphological changes in the flagellar pocket.

The flagellar pocket (FP) is a plasma membrane invagination that forms a subdomain crucial for exocytosis and endocytosis activities. This membranous, flask-like structure is demarcated by cytoskeletal elements, with the basal bodies positioned at one pole and the FPC clinching the FP membrane around the exiting flagellum at the other (Fig. S1). Previous studies have shown that these cytoskeletal structures are essential for FP biogenesis (11).

**FIG 5:**
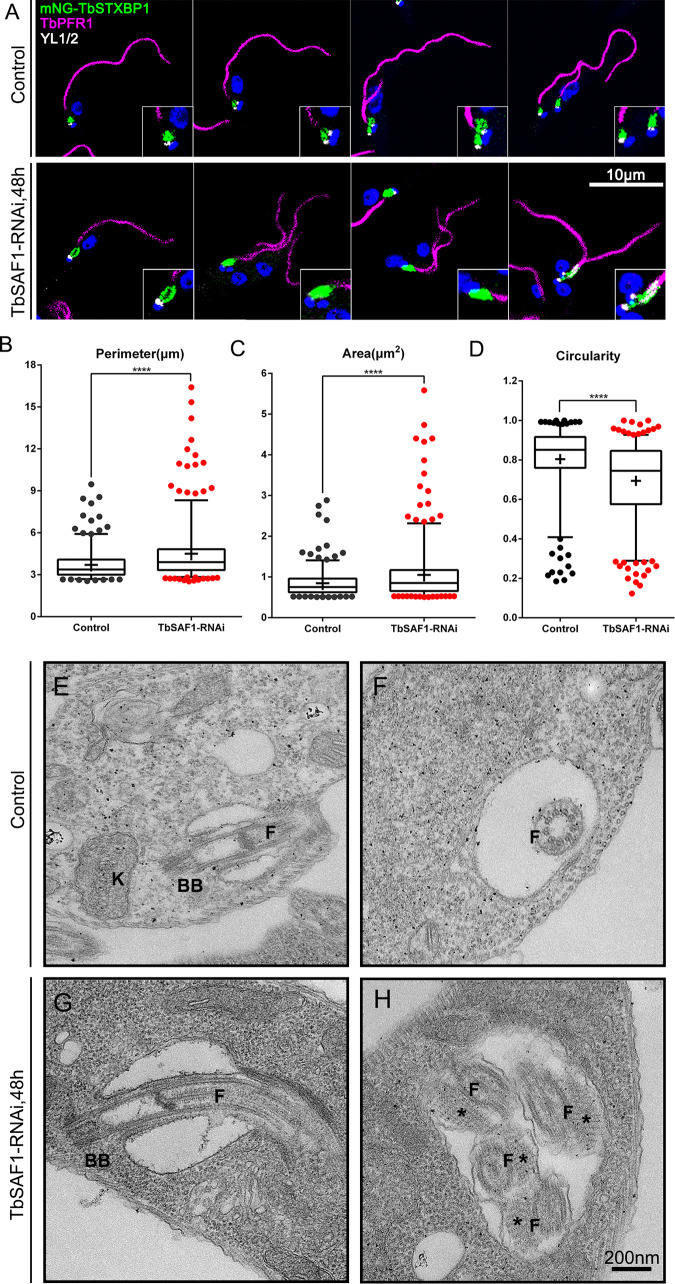
TbSAF1-RNAi generates a larger and elongated flagellar pocket. (A) Cells stably expressing mNG-TbSTXBP1 (green) were induced for TbSAF1-RNAi or not, fixed and costained with YL1/2 for the basal bodies (white) and anti-PFR1 for the PFR (magenta) to ensure identification of the cell cycle stages. (B to D) Perimeter, area, and circularity values were quantified based on the images in panel A. At least 200 cells were counted for each condition. The results are shown as box-and-whisker plots, with the boxes showing the 25th to 75th percentiles, the whiskers showing the 5th to 95th percentiles, and “+” representing the mean. ****, *P* < 0.0001. (E to H) Control (E and F) and TbSAF1-RNAi (G and H) cells were chemically fixed, thin sectioned, and imaged by TEM. Representative cross sections and longitudinal views of the flagellar pockets are shown. BB, basal bodies; K, kinetoplast; F, flagellum; *, PFR.

In TbSAF1-RNAi cells, the MtQ dissociated from the basal bodies, resulting in increased distance between the basal bodies and the FPC. To evaluate whether the flagellar pocket structure may be altered in TbSAF1-RNAi cells, several FP markers were tested, including antibodies directed against FP45 (14) or CRAM (43) and tomato lectin labeled with Texas Red (TL-TR) (44), for their FP labeling. All markers could label the FP area but also suffered from weak signal, heterogeneity, and/or high background levels. Through a search of the Tryptag database (http://tryptag.org/) (36), a putative syntaxin binding protein 1 (TbSTXBP1, Tb927.9.1970) was identified as a potential FP membrane marker. Endogenously expressed mNG-TbSTXBP1 colocalized with TL-TR, sometimes appearing as halos, consistent with the predicted FP membrane localization (Fig. S6). Using mNG-TbSTXBP1 as an FP marker, we showed that the perimeter, area, and circularity of the FP were significantly changed in TbSAF1-RNAi cells compared to control (*P* < 0.0001) (Fig. 5).

10.1128/mBio.00668-20.6 FIG S6 A putative trypanosome homolog of syntaxin binding protein 1 (TbSTXBP1) is an FP marker. Cells with stable, endogenous expression of mNG-TbSTXBP1 were incubated with Texas Red-labeled tomato lectin (TL-TR) at 4°C for 30 min and then fixed with 4% PFA and imaged with a confocal microscope. Note that cell aggregation was observed during treatment with TL-TR. Insets show enlarged views of the FP region, and the line plots demonstrate the colocalization of mNG-TbSTXBP1 and TL-TR in the FP. Download FIG S6, JPG file, 0.8 MB. Copyright © 2020 Dong et al. 2020 Dong et al. This content is distributed under the terms of the Creative Commons Attribution 4.0 International license.

The longer FP in TbSAF1-RNAi cells is most likely due to the increased distance between the basal bodies and the FPC. The larger FP size could be at least partially due to defects in FP segregation. In control cells, two distinct FPs could be observed soon after basal body duplication, and likely before the initiation of new PFR assembly (Fig. 5A, top). As such, all wild-type cells with two flagella also contained two FPs. However, upon TbSAF1-RNAi, ∼35% biflagellated cells contained only one FP focus (Fig. 5A, bottom), suggesting that FP duplication or segregation was affected in these cells.

The FP morphological change upon TbSAF1-RNAi was further confirmed by transmission electron microscopy. Compared to the control (Fig. 5E and F), longer and/or larger FPs sometimes containing more than one flagellum could be observed in TbSAF1-RNAi cells (Fig. 5G and H). Interestingly, the paraflagellar rod (PFR) could be clearly observed in the cross sections of some flagella inside the FP (Fig. 5H), suggesting that the FP had extended beyond the initiation point of PFR along the flagellar axoneme. This is also consistent with our light microscopic observation of position shift for the FPC but not for the PFR (Fig. 3). Note that PFR is never observed in the FP portion of flagellum in a wild-type cell, as the PFR is only assembled after the flagellum exits the FP at the FPC (12).

### Depletion of TbSAF1 does not affect endocytosis.

**FIG 6:**
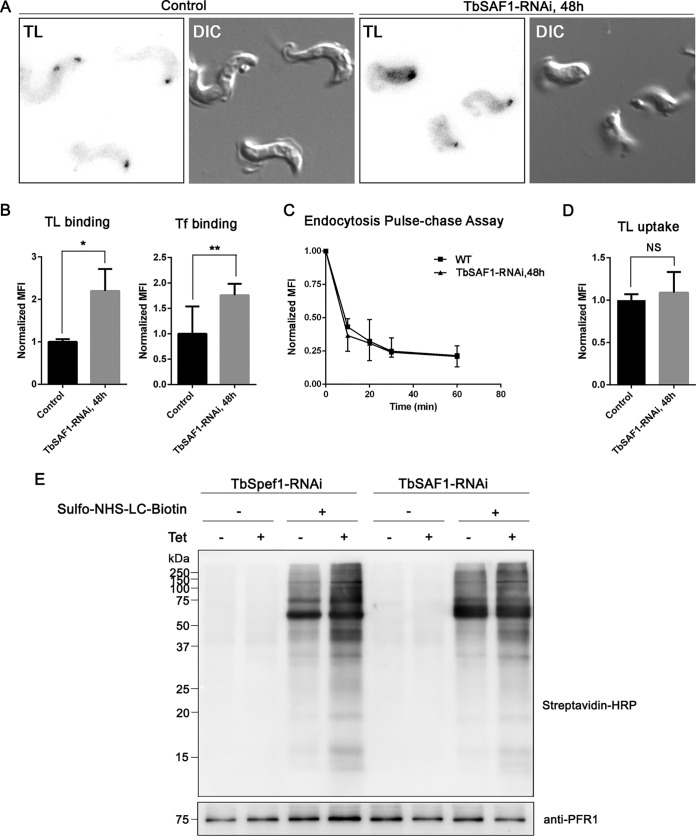
TbSAF1-RNAi does not affect endocytosis but may alter surface protein distribution. (A and B) Control and TbSAF1-RNAi cells were incubated with TL-FITC or Tf-Alexa 633 at 4°C for 30 min, followed by fixation with 4% PFA. Cells were imaged by fluorescence microscopy (A) and/or analyzed by flow cytometry (B). The mean fluorescence intensity (MFI) was normalized against control cells, and the results are shown as means ± the standard deviations (SD) from three independent experiments. (C) The endocytosis of TL-FITC was monitored by flow cytometry of samples taken at 0, 10, 20, 30, and 60 min of incubation at 37°C. The MFIs normalized against *t* = 0 were plotted against incubation time for both control and TbSAF1-RNAi cells. The results are shown as means ± the SD for each condition from three independent experiments. (D) The uptake of TL-TR after 30 min of incubation at 37°C was monitored in control and TbSAF1-RNAi cells by flow cytometry. Normalized MFIs from three independent experiments are shown. (E) Control, TbSpef1-RNAi, and TbSAF1-RNAi cells were incubated with sulfo-NHS-LC-biotin at 4°C. Surface biotinylation patterns were compared by using immunoblots probed with streptavidin conjugated with horseradish peroxidase. Anti-PFR1 was used as a loading control.

FP is essential for endocytosis in T. brucei (45–48). We therefore sought to determine whether endocytosis might be affected in TbSAF1-RNAi cells due to the changes in FP morphology. To address this question, we chose to use bloodstream form T. brucei, the mammal-infectious life cycle stage that has greater endocytosis activity (47–49). Similar to the procyclic-form (PCF) cells, bloodstream-form (BSF) cells depleted of TbSAF1 showed rapid inhibition on cell proliferation, although the cells continued to duplicate at a lower rate (Fig. S7A). A larger FP was also observed by staining with tomato lectin labeled with fluorescein isothiocyanate (TL-FITC) (Fig. 6A) or by TEM (Fig. S7B to E). Multiple flagella, some with associated PFR and occasionally with disorganized axonemes, could be found in these enlarged FPs (Fig. S7E).

10.1128/mBio.00668-20.7 FIG S7 TbSAF1 depletion in bloodstream form cells results in slow growth, FPC, and FP morphological alteration. (A) Bloodstream-form T. brucei were induced for TbSAF1-RNAi and cell proliferation was measured every 24 h. Slower but continuous cell proliferation was observed upon depletion of TbSAF1, similar to the procyclic cells. (B to E) Control and TbSAF1-RNAi cells were chemically fixed, thin sectioned, and imaged by TEM. Representative longitudinal and cross-section views of the FPs are shown. Insets show the enlarged views of the boxed regions. BB, mature basal body; pBB, probasal body; K, kinetoplast; F, flagellum; *, PFR. Download FIG S7, JPG file, 2.8 MB. Copyright © 2020 Dong et al. 2020 Dong et al. This content is distributed under the terms of the Creative Commons Attribution 4.0 International license.

Endocytosis was evaluated with pulse-chase experiments (Fig. 6; Fig. S8), where control and TbSAF1-RNAi cells were incubated with TL-FITC at 4°C to facilitate binding to the FP without internalization. The cells were then washed to remove unbound TL-FITC and shifted to 37°C to initiate endocytosis. Since TL-FITC fluorescence is sensitive to low pH, endocytosed TL-FITC gradually loses fluorescence as it enters endosomes and lysosomes with increasing acidity (50, 51). Interestingly, more TL-FITC was bound to TbSAF1-RNAi cells at *t* = 0 of the pulse-chase compared to the control (Fig. 6A and B). Increased binding was also observed with transferrin (Fig. 6B). However, the rate of endocytosis, as monitored by the loss of TL-FITC fluorescence using flow cytometry and microscopy, was similar in control and TbSAF1-RNAi cells (Fig. 6C; Fig. S8). Consistently, the uptake of TL-TR (i.e., the accumulation of intracellular TL over a 30-min incubation at 37°C) was similar between control and TbSAF1-RNAi cells (Fig. 6D).

10.1128/mBio.00668-20.8 FIG S8 Effect of TbSAF1-RNAi on endocytosis. Control and TbSAF1-RNAi cells were stained with TL-FITC for 30 min at 4°C and pulse-chased in serum-free medium at 37°C for the indicated times. Cells were fixed with 4% PFA and imaged with a fluorescence microscope (A) or analyzed by flow cytometry (B). Representative flow cytometry results, as shown in panel B, were quantitated and are shown in Fig. 6. Download FIG S8, JPG file, 2.0 MB. Copyright © 2020 Dong et al. 2020 Dong et al. This content is distributed under the terms of the Creative Commons Attribution 4.0 International license.

The increased binding of endocytosis markers may be due to the larger FPs in TbSAF1-depleted cells. In addition, increased binding of TL or transferrin (Tf) to the cell membrane was also observed in TbSAF1-RNAi cells (Fig. 6A), suggesting possible changes in the cell surface besides the flagellar pockets. To further test this possibility, control and TbSAF1-RNAi cells were incubated with sulfo-NHS-LC-biotin (Thermo Fisher Scientific, Inc.) at 4°C to allow biotinylation of surface proteins. Enhanced surface biotinylation and/or more biotinylated proteins were found in TbSAF1-RNAi cells compared to control (Fig. 6E). Similar changes in surface biotinylation pattern were also found in TbSpef1-RNAi cells, indicating that TbSpef1 and TbSAF1 both affect cell surface organization, probably through the same pathway.

## DISCUSSION

Our BioID screening revealed a complex interaction network formed by TbSpef1. Of the eight novel BioID candidates characterized (Fig. 1), two are present on the basal bodies (Tb927.7.4370 and Tb927.6.1220), three are present in the HC/bilobe region (Tb927.10.12720, Tb927.5.570, and Tb927.3.2760), and three are present at the TbSpef1-marked MtQ (Tb927.8.8160, Tb927.10.250, Tb927.11.1220), highlighting the close association of the MtQ with the basal bodies and the HC/bilobe. Further characterization of the MtQ-basal body association revealed a critical role for this association in cytoskeletal organelle positioning and membranous FP structure in T. brucei (Fig. 2–6). Like Spef1/CLAMP previously characterized in vertebrates, TbSpef1 can also bind and bundle microtubules (Fig. S1), attesting to this evolutionarily conserved molecular function from protozoa to mammals.

Spef1/CLAMP has been implicated in various microtubule-related functions. In mice, Spef1 is present on the central pair and is required for the central pair biogenesis and ciliary beating (4). In *Xenopus*, Spef1/CLAMP is involved in at least two developmental functions: (i) the directed movement of invasive subapical layer toward the epidermis of the embryo skin, during which Spef1/CLAMP recruits stable, acetylated MTs to the apical PAR complex (5), and (ii) the planar cell polarity (PCP) pathway (6), during which Spef1/CLAMP is required for proper PCP protein localization and polarized organization of MTs (52). In Leishmania mexicana, Spef1 is present on microtubules associated with lysosome tubules (29, 53). Together with our work on TbSpef1, these studies suggest diverse, cell-specific functions of Spef1/CLAMP that may be achieved via its conserved microtubule activities and its diverse molecular interaction network in different cells.

**FIG 7:**
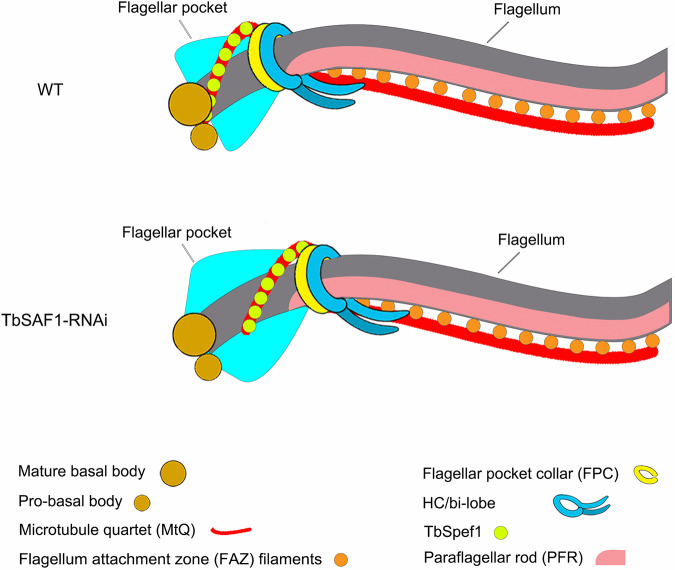
Schematic representation of the misplaced cytoskeletal organelles in cells with TbSAF1 depletion. Upon TbSAF1-RNAi, TbSpef1-MtQ is dissociated from the basal bodies, leading to distal shift of MtQ-associated FPC, HC/bilobe, and FAZ filament. The flagellar pocket is morphologically altered concomitantly.

T. brucei contains a wealth of single-copied organelles that are tethered together to form one continuous network, facilitating their coordinated duplication and segregation during the cell cycle (14, 15, 54). The basal bodies seem to lie at the center of this network, forming two cytoskeletal modules. In one module, the basal bodies directly nucleate the flagellum and are physically linked to the kinetoplast through the TAC. In the other module, the MtQ originates from a region between the two basal bodies, and the MtQ is associated with the FPC, the HC/bilobe, and the FAZ filament. During the cell division cycle, the biogenesis of the two cytoskeletal modules are highly coordinated, possibly via the action of cell cycle regulators that can move between the two cytoskeletal modules, such as polo-like kinase 1 (55). Depletion of TbSAF1 severed the MtQ-basal body association, consequently disconnecting these two cytoskeletal modules (Fig. 7). The duplication and segregation of each of the cytoskeletal module was not obviously affected, although subtle changes in their coordination may result in the slower cell proliferation observed in TbSAF1-RNAi cells.

Although both are microtubule structures, the flagellar axoneme and the MtQ are associated with the basal bodies in distinct manners. The flagellum axonemal microtubules are direct outgrowth from the basal bodies (56–59). The MtQ-basal body association, however, is similar to the interaction between the spindle microtubules and the centrosomes in higher eukaryotes. The “-” ends of the spindle microtubules are cross-linked and bundled through the activities of microtubule-associated proteins and microtubule motors, and converged to form spindle poles, which are then anchored to the centrosomes via interaction with centrosomal components (60–62). Similarly, TbSpef1 can also bind and bundle microtubules (Fig. 1) and anchor the MtQ to the basal bodies via direct or indirect interaction with TbSAF1. How TbSpef1, a microtubule binding protein, is specifically enriched in the MtQ between the basal bodies and the FPC, and whether microtubule motor proteins play a role in this specific localization remains to be determined.

Our results also emphasized the critical functions of the cytoskeletal elements in shaping the flagellar pocket (FP). Without subpellicular microtubules surrounding the FP membrane (Fig. S1A), the FP serves as the exclusive site for endocytosis in T. brucei (46–48, 63). Structurally, the FP is bounded by two important cytoskeletal elements, the basal bodies on one pole and the FPC on the other; both form tight junctures with FP membranes (63). Dissociation of the MtQ and the FPC from the basal bodies during SAF1-RNAi changed the FP structure (Fig. 5), demonstrating the essential role of the two poles and their connecting TbSpef1-MtQ in shaping the FP. Our results also identified and/or confirmed four new MtQ markers (Tb927.8.8160, Tb927.10.250, Tb927.11.1220, and Tb927.4.4180). These new MtQ markers will provide useful tools to further investigate the role of MtQ and its functions in FP organization. Recent studies in Leishmania mexicana demonstrate a role of FAZ in FP architecture (64). In T. brucei, however, inhibition of the FAZ filament by TbCC2D silencing had little effect on FP organization (26), while the MtQ seems to have a more specific role in shaping the FP. Curiously, despite the identification of several HC/bilobe proteins as TbSpef1-BioID candidates (Table 1), no FPC proteins have been found in any of the three independent BioID experiments. The TbSpef1-MtQ may form more extensive interactions with proteins in the HC/bilobe region than the FPC, contributing to the tripartite interaction between the FPC, the MtQ, and the HC/bilobe (16).

A surprising finding of this study was the lack of obvious endocytic defects in cells depleted of TbSAF1, despite altered FP morphology and inhibited biogenesis. Growth delay but not complete growth inhibition was observed in both procyclic and bloodstream-form cells lacking TbSAF1 (Fig. S4; Fig. S7). This is distinct from endocytosis mutants, such as clathrin-RNAi, that exhibited rapid cell swelling and death (46). Our results suggest that an alteration in FP structure alone is probably not sufficient to inhibit its endocytic function. TbSAF1 depletion may also lead to changes in surface proteins (Fig. 6). Since trypanosome surfaces are highly organized to evade host immune attack, changes in parasite surface protein organization may result in increased susceptibility to host immune attacks and parasite clearance. Whether and how FP structure affects parasite surface organization remains to be investigated.

## MATERIALS AND METHODS

### Cells and cultures.

The T. brucei procyclic-form (PCF) 29-13 cell line (65) was maintained at 28°C in Cunningham medium. The bloodstream-form (BSF) tetracycline-cumate dual inducible cell line DIb427 (66) was maintained in HMI-9 medium at 37°C with 5% CO_2_. Both media contain 10% heat-inactivated fetal bovine serum (HyClone, Inc.) and are supplemented with 15 μg/ml G418 and 50 μg/ml hygromycin for PCF or 2.5 μg/ml puromycin for BSF cultures. Stable transfections were performed by electroporation, and clonal cell lines were selected and maintained with appropriate antibiotics as previously described (67).

### Cell growth assays.

PCF cells were maintained in log phase with densities between 5 × 10^5^ and 1 × 10^7^ cells/ml through dilution with fresh medium daily, whereas BSF cells were kept at between 1 × 10^5^ and 2 × 10^6^ cells/ml, also at exponential phase. To perform growth assays, the cell density was measured using a hemocytometer every 24 h prior to dilution to 5 × 10^5^ cells/ml for PCF or 1 × 10^5^ cells/ml for BSF. The cell doubling number was calculated as follows: doubling number = log_2_(*N_t_* × DF/*N*_0_), where *N_t_* represents the cell density at each time point, *N*_0_ is the initial cell density at *t* = 0, and DF is the cumulative dilution factor for each time point.

### Molecular methods.

Tetracycline-inducible TbSpef1-RNAi cell line was described previously (14). To generate the tetracycline-inducible TbSAF1-RNAi cell line, a fragment of TbSAF1 cDNA (nucleotides 3004 to 3493) selected by using RNAit (https://dag.compbio.dundee.ac.uk/RNAit/) (68) was subcloned into the XbaI site in the p2T7-177 plasmid (69). For endogenous tagging, a modified pCR4Blunt-TOPO-vector (18) or the pPOT6 series vectors (70) were used following previously published procedures.

### *In vitro* microtubule binding and bundling assays.

Microtubules (MTs) were polymerized *in vitro* according to the manufacturer’s instructions (Cytoskeleton, Inc.). For the MT binding assay, varied concentrations of purified His-tagged TbSpef1 proteins were incubated with *in vitro*-assembled and paclitaxel-stabilized MTs at room temperature for 30 min. The reaction mix was then diluted with microtubule cushion buffer (80 mM PIPES [pH 7.0], 1 mM MgCl_2_, 1 mM EGTA, 60% glycerol) supplemented with 20 μM paclitaxel and centrifuged at 100,000 × *g* and 30°C for 30 min. The supernatants were carefully transferred into new Eppendorf tubes, and equal fractions of the pellets and the supernatants were analyzed by immunoblotting. Purified His-TbSpef1 without MTs were used as a negative control.

For MT bundling assays, varied concentrations of purified His-TbSpef1 were incubated with rhodamine-labeled and paclitaxel-stabilized MTs at room temperature and imaged immediately by fluorescence microscopy.

### Proximity-dependent biotin identification (BioID).

Cells with stable endogenous expression of Myc-BirA*-TbSpef1 were cultivated in the presence of 50 μM biotin for 24 h. Wild-type 29-13 cells were used as negative control. Totals of 1 × 10^9^ to 2 × 10^9^ cells for each sample were harvest by centrifugation at 3,000 rpm for 7 min (Eppendorf 5810). According to a published method (32), the cells were washed three times with phosphate-buffered saline (PBS) and lysed in 5 ml of ice-cold PBS supplemented with 1% (vol/vol) NP-40 and protease inhibitors. The cell lysates were centrifuged at 3,400 × *g* for 10 min at 4°C. The pellet containing NP-40-resistant cytoskeleton was solubilized in 1 ml of lysis buffer containing 0.4% sodium dodecyl sulfate (SDS), 500 mM NaCl, 5 mM EDTA, 1 mM dithiothreitol, 50 mM Tris-HCl (pH 7.4), and protease inhibitors. The mixture was then incubated at room temperature for 30 min and centrifuged at 12,000 × *g* for 10 min. The supernatant was carefully transferred to an Eppendorf tube containing streptavidin-coupled Dynabeads (Invitrogen), followed by incubation at 4°C overnight with gentle rotation. A magnetic stand was used to separate and wash the beads as follows: twice with 1 ml of PBS containing 1% SDS, twice with 1 ml PBS containing 1% NP-40, and twice with 1 ml of PBS only. After washing, the beads were transferred to 200 μl triethylammonium (500 mM pH 8.5) containing 2 mM TCEP [Tris(2-carboxyethyl)phosphine hydrochloride]. The solution was agitated for 1 h at 65°C to reduce the disulfide bond of the proteins. To alkylate the suspension, 4 mM methyl methanethiosulfonate (MMTS) was added, followed by further incubated at room temperature for 15 min. Trypsin digestion (2.5 μg of trypsin for each sample) was performed at 37°C overnight. After digestion, the magnetic beads were removed, and the peptide solution was subjected to LC-MS/MS analyses. Proteomics data were analyzed with MASCOT Server 2.4.0 (Matrix Science) and the T. brucei protein database (TbruceiTREU927, release 28) as previously published (71). The identification threshold was set at a 1% false discovery rate, and the estimation of absolute protein amount—the “protein content”—was calculated based on the exponentially modified protein abundance index (emPAI) (72).

### Fluorescence labeling and microscopy methods.

T. brucei cells attached to coverslips were fixed with 4% paraformaldehyde (PFA) in PBS for 15 min and permeabilized with 0.25% Triton X-100 in PBS for 5 min. For cytoskeleton preparations, cells were extracted with 0.25% NP-40 in PEM buffer containing 100 mM PIPES [piperazine-*N*,*N′*-bis(2-ethanesulfonic acid)] (pH 6.9), 1 mM EGTA, and 1 mM MgSO_4_, followed by 4% PFA fixation. Cell fixation and permeabilization can also be performed using methanol at –20°C for 5 min.

For immunofluorescence microscopy, anti-TbSAS6 (1:500) (41), YL1/2 (1:2,000; AbCam), anti-BILBO1 (1:4,000) (11), anti-CC2D (1:5,000) (26), anti-PFR2 (1:5,000; Abcam), anti-TbSpef1 (1:500) (14), anti-MORN1 (1:5,000) (32), and anti-Centrin4 (1:500) (17) antibodies were used to label the basal bodies, flagellar pocket collar, FAZ filament, paraflagellar rod, proximal segment of MtQ between the basal body and flagellar pocket collar, and bilobe or bilobe with basal bodies, respectively.

To label the flagellar pockets, PCF or BSF cells were incubated with Texas Red- or FITC-conjugated tomato lectin (TL; Vector Laboratories, Inc.) at 4°C for 30 min. TL-labeled cells were washed thoroughly with ice-cold gPBS (PBS containing 1% [wt/vol] glucose) before fixation with 4% PFA at 4°C for 20 min (73). The fixed cells were then washed once with PBS to remove PFA and attached to polylysine-coated coverslips by centrifugation.

Fluorescence images were acquired either by using a Zeiss Axio Observer inverted microscope equipped with a CoolSNAP HQ2 CCD camera (Photometrics) or a Fluoview FV3000 (Olympus, Inc.) confocal microscope equipped with a U Plan Super Apochromat 60×/1.35 objective. 3D-SIM images were acquired using a Nikon Ti-E motorized inverted microscope with a perfect focus system. All images were processed and quantitated using ImageJ and appropriate plugins. Figures were prepared using either Adobe Photoshop or Adobe Illustrator.

### Electron microscopy methods.

Electron microscopy analyses were performed based on previously established methods (19, 74). For negative staining, approximately 5 × 10^7^ cells were harvested and resuspended with 500 μl of cultivation medium without serum. Cell suspension was placed on plasma cleaned grids and incubated for at least 15 min to allow cell attachment. The grids were then transferred sequentially to droplets of PEME buffer (100 mM PIPES [pH 6.9], 1 mM MgSO_4_, 0.1 mM EDTA, 2 mM EGTA) containing 1% (vol/vol) NP-40 at room temperature and PEME buffer containing 1% (vol/vol) NP-40 and 1 M KCl at 4°C and then extracted for 5 min each. The grids were then rinsed in PEME buffer for four times and fixed in PEME buffer containing 2.5% glutaraldehyde for 5 min at room temperature. After the grids were rinsed in double-distilled water, they were quickly placed in a drop of 0.5% gold thioglucose and immediately drained over a piece of filter paper. The grids were air dried for 5 to 10 min before visualization with a 120-kV Tecnai T12 electron microscope (FEI).

For chemical fixation, ∼2 × 10^8^ cells were prefixed *in situ* by mixing the cell culture with 1/10 volume of 25% glutaraldehyde. The prefixed cells were then fixed in 1 ml of buffered fixative (2.5% final glutaraldehyde, 2% paraformaldehyde, and 100 mM phosphate buffer [pH 7.0]) at 4°C overnight. For BSF cells, 50 mM sucrose was also added into the buffered fixative to maintain cell morphology. After postfixation with 1% osmium and UranyLess (EMS, Inc.) staining, the cells were dehydrated through a series of increasing concentrations of acetone: 30, 50, 70, and 90% and then three times 100%, followed by incubation with 100% propylene oxide and substitution with epoxy resin (Araldite, Inc.). Cells were embedded in Epoxy resin at 60°C for at least 48 h.

To image the entire flagellar pocket region containing the basal bodies and the MtQ, five continuous sections (each 150 to 200 nm thick) encompassing the flagellar pocket regions were generated from the chemically fixed control and TbSAF1-RNAi cells by using an Ultracut UCT ultramicrotome (Leica). Each section was imaged by tilting it around two orthogonal axes on a Tecnai T12 (FEI). Separate tomograms (a total of 10 tomograms for each sample) were computed from each tilt series for each section; these were then aligned and combined to produce a single tomogram using IMOD (75). Features of the basal bodies, the flagellar pocket, and the MtQ were manually annotated, and three-dimensional models were reconstructed using IMOD (75).

### Binding, endocytosis, and uptake analyses.

For binding analyses, ∼2 × 10^7^ BSF control and TbSAF1-RNAi (48 h postinduction) cells were harvested by centrifugation at 800 × *g* for 10 min. Binding assays with TL were modified from previous methods (51). Briefly, cells were washed with ice-cold serum-free HMI9 medium supplemented with 0.5 mg/ml bovine serum albumin (BSA) and then resuspended in the same buffer, followed by incubation on ice for 15 min. TL labeled with FITC (TL-FITC) was then added to the cells to a final concentration of 5 μg/ml, followed by incubation on ice for another 30 min to allow binding. Cells were washed thoroughly with ice-cold serum-free HMI9 medium containing 0.5 mg/ml BSA and fixed with 4% PFA at 4°C. Tf binding was performed as previously published (76). In brief, cells were washed with HEPES buffered-saline supplemented with 1% (wt/vol) glucose and preincubated with HMI9 medium containing 0.5 mg/ml BSA for another 2 h to deplete Tf in the medium. The cells were then washed with ice-cold gPBS, incubated with 25 μg/ml Alexa 633-conjugated Tf (Thermo Fisher Scientific, Inc.) for 30 min, washed with gPBS, and fixed with 4% PFA. Binding was monitored by fluorescence microscopy or flow cytometry using a CytoFLEX LX flow cytometer (Beckman Coulter, Inc.).

For endocytosis, binding to TL-FITC was performed on ice for 30 min as described above. After the washes with ice-cold HMI9 medium containing 0.5 mg/ml BSA, the cells were transferred to 37°C to initiate endocytosis. Equal amounts of sample were taken at *t* = 0, 10 min, 20 min, 30 min, and 60 min, and immediately fixed with 4% PFA. After fixation, half of the samples were attached to the coverslips for immunofluorescence microscopy, and the other half were analyzed by flow cytometry. The flow cytometry data were processed by using CytExpert 2.1 analysis software (Beckman Coulter, Inc.).

For TL-FITC uptake analyses, the cells were harvested and washed as described above. Upon the addition of TL-FITC, the cells were transferred to 37°C to initiate uptake. After incubation for 30 min, the cells were fixed with 4% PFA and analyzed by flow cytometry.

### Surface biotinylation.

Approximately 2 × 10^8^ cells were harvested and washed thoroughly with ice-cold gPBS. The cells were incubated with or without 1.5 mg/ml freshly prepared sulfo-NHS-LC-biotin (Thermo Fisher Scientific) on ice for 30 min. Then, 100 mM Tris-HCl (pH 6.8) was added to stop the biotinylation/cross-linking reaction. The cells were washed twice with gPBS containing 100 mM Tris-HCl (pH 6.8) before lysis with SDS loading buffer and analysis by immunoblotting.
